# Knowledge and utilization of sexual and reproductive healthcare services among Thai immigrant women in Sweden

**DOI:** 10.1186/s12914-016-0100-4

**Published:** 2016-10-10

**Authors:** Eva Åkerman, Per-Olof Östergren, Birgitta Essén, Cecilia Fernbrant, Ragnar Westerling

**Affiliations:** 1 Division of Social Medicine, Department of Public Health and Caring Sciences, Uppsala University, Box 564, 751 22 Uppsala, Sweden; 2 Division of Social Medicine and Global Health, Department of Clinical Sciences Malmo, Lund University, Jan Waldenströms gatan 35, Malmö, 205 02 Sweden; 3 Department of Women’s and Children’s Health, International Maternal and Child Health, Uppsala University, Akademiska sjukhuset, Uppsala, 751 85 Sweden

**Keywords:** Social capital, Immigration, Thai women, Healthcare utilization, HIV/STI

## Abstract

**Background:**

Migration from Thailand to Sweden has increased threefold over the last 10 years. Today Thailand is one of the most common countries of origin among immigrants in Sweden. Since the year 2000, new HIV cases are also more prevalent among Thai immigrants compared to other immigrant nationalities in Sweden. The purpose of this study was to investigate the association between knowledge and utilization of sexual and reproductive healthcare services, contraceptive knowledge and socio-demographic characteristics and social capital among Thai immigrant women in Sweden.

**Methods:**

This is a cross-sectional study using a postal questionnaire to all Thai women (18–64) in two Swedish regions, who immigrated to the country between 2006 and 2011. The questionnaire was answered by 804 women (response rate 62.3 %). Bivariate and multivariate logistic regression analyses were used.

**Results:**

The majority (52.1 %) of Thai women had poor knowledge of where they should turn when they need sexual and reproductive healthcare services. After controlling for potential confounders, living without a partner (OR = 2.02, CI: 1.16–3.54), having low trust in others (OR = 1.61, CI: 1.10–2.35), having predominantly bonding social capital (OR = 1.50, CI: 1.02–2.23) and belonging to the oldest age group (OR = 2.65, CI: 1.32–5.29) were identified as risk factors for having poor knowledge. The majority (56.7 %) had never been in contact with healthcare services to get advice on contraception, and about 75 % had never been HIV/STI tested in Sweden. Low utilization of healthcare was associated with poor knowledge about healthcare services (OR = 6.07, CI: 3.94–9.34) and living without a partner (OR = 2.53, CI: 1.30–4.90). Most Thai women had knowledge of how to prevent an unwanted pregnancy (91.6 %) and infection with HIV/STI (91.1 %).

**Conclusions:**

The findings indicate that social capital factors such as high trust in others and predominantly bridging social capital promote access to knowledge about healthcare services.

However, only one-fourth of the women had been HIV/STI tested, and due to the HIV prevalence among Thai immigrants in Sweden, policy makers and health professionals need to include Thai immigrants in planning health promotion efforts and healthcare interventions.

## Background

Providing equitable access to healthcare services is a human rights concern and a global challenge. Access to healthcare services is also one of the important determinants of a population’s health. The World Health Organization defines health as “a state of complete physical, mental and social wellbeing, and not merely the absence of disease or infirmity” [1]. In line with this definition, sexual and reproductive health and rights mean that people are able to have a responsible, satisfying and safe sex life, that they have the capability to reproduce and the freedom to do so, and that they are also able to gain information about and have access to safe, effective, affordable and acceptable methods of contraception and access to prevention, treatment and care for sexually transmitted infections, including HIV/AIDS.

According to Swedish law, all residents of Sweden have the right to good health and healthcare on equal terms [2]. However, previous studies have shown that access to and use of healthcare services are associated with numerous variables, such as socioeconomic factors and occupation level [3–5], ethnicity [6–8] and the behaviour of the healthcare providers [9–12]. A lack of knowledge about health and the healthcare system among immigrants can limit access to healthcare or explain why they use healthcare inappropriately [13–15]. Immigration status is another predictor of access to and use of healthcare. Immigrants coming to Sweden as refugees or asylum-seekers are entitled to a health examination, which is offered free of charge. The right to this health examination is explicitly stated in Swedish law (2008:344) [16]. The intention is to identify infectious diseases such as HIV/AIDS and TB or other health problems. A further intention is to introduce refugees and asylum-seekers to the Swedish healthcare system and inform them of how to contact healthcare and dental care [17]. People who immigrate to Sweden for other reasons, such as partnership and marriage with Swedish citizens, are not covered by this law and therefore normally not offered this free health examination, although they constitute a large group among the total number of immigrants. According to Swedish health policy, primary healthcare is the first place for people to turn when they seek healthcare. Patients who want to see a specialist must get a referral from primary care. A visit to a doctor at the primary care level costs about 200 SEK (20 USD) for all residents of Sweden regardless of nationality. Swedish healthcare policy also includes several screening programmes, such as the HIV/Chlamydia trachomatis test for pregnant women and the cervical cancer screening program for all women aged 23–60. Women are called to an appointment for a cervical cancer screening every third year up to the age of 50 and every fifth year thereafter. Women or men who want advice on contraceptives are advised to visit maternal healthcare, which also serves pregnant women with routine health checks during their pregnancy.

Several studies have shown the positive associations between social capital and health outcomes [18–23], measured as self-rated health and mental health. Social capital has been defined in different ways, and no shared definition among disciplines has been developed. In summary, concepts of bonding and bridging social capital include trust in others, social participation, networks and shared norms and values with racially or ethnically similar and dissimilar people [19, 24–27]. Some of the mechanisms that explain the associations between social capital and health outcomes are related to information, knowledge and access to services. Social capital develops channels for the distribution of knowledge and information [28], and social relationships provide information, which in turn can serve as a basis for action [25]. Berkman et al. point out that participation in networks can provide access to material resources such as good health services and jobs [29]. Rostila found that immigrants living in Sweden with homogenous and closed social networks have poorer self-rated health than immigrants who interact in networks with a higher proportion of native Swedes [30]. Studies of how social capital influences knowledge and use of healthcare services are more limited.

Sweden is a multicultural society, and approximately 15 % of the total population are foreign born. Migration from Thailand to Sweden has increased threefold over the last 10 years. Today, Thailand is one of the most common countries of origin among immigrants in Sweden. In 2014, there were 38,129 persons born in Thailand who were registered in Sweden [31]. Approximately 80 % of Thai-born people in Sweden are women. Of these women and those that have a relationship, almost 80 % are married to Swedish men. The increasing number of women migrating abroad because they marry men from another country is an international phenomenon, with an over-representation of South–north migration flows of females from Southeast Asia, Eastern Europe and Russia [32]. However, one study conducted in Sweden describes Thai women as active agents in their migration process [33]. Since the year 2000, new HIV cases are also more prevalent (in absolute numbers) among Thai immigrants compared to other immigrant nationalities in Sweden [34]. Despite this, and the growing number of Thai women in Sweden, little is known about their knowledge of the Swedish healthcare system and their utilization of healthcare services. To our knowledge, there are no routine preventive interventions directed towards the Thai group. Previous research has shown that many immigrants in high-income countries come late for their first HIV test, so called late presenters [35, 36]. As a consequence they are diagnosed at a stage when treatment is normally already applied. In year 2013 in Sweden, approximately 6,200 people were living with HIV, and 3,666 (about 60 %) were immigrants. More than 60 % of all newly diagnosed HIV cases in 2011 were late presenters, and the majority of these were immigrants [37]. During the past decade, about 400 cases of new HIV diagnoses are reported annually, and among these approximately 75 % are immigrants, which is the highest percentage of new HIV diagnoses among immigrants in the EU countries [38]. During the period 2009–2013, a total of 189 Thais tested HIV positive, followed by Eritreans with 152 HIV cases and Ethiopians with 134 cases. However, seen in proportion to the total number of Thai, Eritreans and Ethiopians migrating to Sweden during 2009–2013, the Ethiopians had a mean prevalence rate of 4.2 %, which was higher compared to the Eritreans (1.9 %) and Thais (1.8 %).

Since Thai women coming as presumptive partners are not offered a free health examination, as refugees or asylum-seekers are, it is relevant to investigate their knowledge and use of Swedish healthcare services. The purpose of this study was to investigate the association between knowledge and utilization of sexual and reproductive healthcare services, contraceptive knowledge and socio-demographic characteristics and social capital among Thai immigrant women in Sweden.

## Methods

### Study setting

The study was conducted in two regions: Skåne and Sjuhäradsbygden. Skåne is the most southern region of Sweden and consists of 33 municipalities with a total population of approximately 1,288,900; of which 19 % are foreign-born and 1.77 % estimated to be of Thai origin [31]. The municipalities are situated in both rural areas and areas close to larger cities in Sweden, Lund and Malmö, each with a university hospital. Sjuhäradsbygden is a rural area located in the southwest of Sweden and has seven municipalities comprising approximately 185,000 inhabitants, of whom 15 % are foreign-born and around 1.89 % are of Thai origin.

### Study design

A cross-sectional study that used a postal questionnaire was conducted in the spring of 2012 in two regions in Sweden, Skåne and Sjuhäradsbygden. The study population consisted of all Thai women aged 18–64 years (*n* = 1291) who were born in Thailand, immigrated to Sweden between 2006 and 2011 and were registered in Skåne or Sjuhäradsbygden. Data collection was done in collaboration with Statistics Sweden (SCB: Statistiska centralbyrån), which distributed and administered the questionnaires. In total, 1,291 Thai women were listed in the Total Population Register (RTB) and received a public health questionnaire in the Thai language by ordinary mail. RTB is a national register of all Swedish citizens, managed by Statistics Sweden. Each respondent received an accompanying letter explaining the purpose of the study, that participation was voluntary and that their answers would remain anonymous. All respondents, except those who had already answered the questionnaire, received three reminders by postage mail. The response rate was 62.3 %. The research project was approved by the Regional Ethical Review Board at Lund University, registration number 2011/521.

### Questionnaire

The questionnaire consisted of 98 questions covering the following themes: socio-demographic issues, self-rated somatic and mental health, use and knowledge of healthcare services, exposure to violence, social capital including trust and social participation, lifestyle factors such as alcohol and tobacco use, and nutrition and physical activity.

The majority of the 98 items were derived from several other comprehensive public health questionnaires used in Sweden [39]. The questionnaire used in the case here was tested in focus group discussions with Thai women in order to ensure its cultural pertinence. The questionnaire was initially written in Swedish, thereafter translated into Thai and then independently back-translated into Swedish. It was pilot tested and revised before being finalized.

### Survey measures

#### Outcome variables

Four outcome variables were used in this study.

##### Contraceptive knowledge

Two direct pertinent questions based on self-reported knowledge, similar instruments also used in other studies related to sexual and reproductive health [40–42], were applied to measure contraceptive knowledge: 1) “Do you know how to protect yourself from an unwanted pregnancy?” 2) “Do you know how to protect yourself from sexually transmitted infections (HIV/STI)?” Possible answers to both questions were “yes” and “no”. Respondents who answered yes were classified as having knowledge. Those who answered no were classified as having a lack of knowledge.

##### Knowledge about healthcare

3) Knowledge about the Swedish sexual and reproductive healthcare service was based on four questions related to respondents’ perceived knowledge about the Swedish sexual and reproductive healthcare services: “Do you know where to go if you want to take a chlamydia/HIV/hepatitis test (HIV/STI)”, a similar item is widely used for monitoring of national HIV/AIDS programs in many countries worldwide [43]. The following items were developed for the purpose of this study: “Do you know where to go if you get pregnant and are considering getting an abortion?”, “Do you know where to go if you want advice on contraception?” and “Do you know where to go if you want to get a gynaecological screening?”. The response options were “yes (=1)” and “no (=0)”, with the score ranging from 0 to 4. For the purpose of logistic regression analyses, the aggregated scores were dichotomized into “good” and “poor” knowledge. Those who scored at least 3 were considered to have good knowledge and the remaining were considered to have poor knowledge.

##### Utilization of healthcare

4) Utilization of sexual and reproductive healthcare services consisted of three questions, which have been used in various health survey instruments worldwide [43–47]: “Have you ever been in contact with healthcare services in Sweden to get a gynaecological screening?”,”Have you ever been in contact with healthcare services in Sweden to get advice on contraception?”, and “Have you ever been in contact with healthcare services in Sweden to get tested for chlamydia/HIV/hepatitis (HIV/STI)?”. The responses for these three questions were “yes (=1)” and “no (=0)”, with a score ranging from 0 to 3. For the purpose of data analysis, respondents’ summary scores were dichotomized into “low” or “high/average” utilization. Respondents who answered that they never used some of the reproductive services or used only one of the services were classified as having low utilization and the remaining were classified as having high/average utilization.

#### Explanatory variables

##### Socio-demographic variables

The socio-demographic variables were age, marital status, education level, immigration year and lacking cash reserves (not being able to get hold of 15,000 SEK (1500 USD) within a week if needed). Age was divided into three groups: 18–30 years old, 31–45 years old and older than 46 years. Marital status was dichotomized as living apart/cohabiting/married or single/divorced/widow. Education was grouped into four levels: 0–6 years, 7–9 years, 10–12 years and more than 12 years. Immigration year, being the year the women received residence permits in Sweden, was categorized into three groups: 2006–2007, 2008–2009 and 2010–2011. Lacking cash reserves was based on the question: “If you suddenly end up in an unforeseen situation, would you be able to get hold of 15,000 SEK within a week if needed?”. Possible answers were “yes” and “no.”

##### Social capital variables

Social capital variables included trust in others, bonding vs. bridging trust, bonding vs. bridging relationships and social participation. Our definitions of bonding vs. bridging social capital factors were based on previous studies in which bonding and bridging were defined on the basis of relationships with ethnically homogeneous or heterogeneous people [19, 26, 27, 48]. Trust in others included four questions, and it has been widely used in studies related to health and sexual and reproductive health and rights [24, 48, 49]; “most people would take advantage of you if they had an opportunity”, “most people try to be fair”, “you can trust most people” and “you cannot be too careful when dealing with other people”. Possible answers were “I do not agree at all”, “I do not agree”, “I agree” and “I agree completely”, given the values of 1 to 4, respectively, meaning a maximum score of 16. Based on the median, respondents who scored below (<10) were considered to have “low trust” and those that scored above the median (>10) were considered to have “high trust”.

Bonding vs. bridging trust was measured by the question “Does a person’s background (e.g. sex, education or country of origin) affect your level of trust of them, for example the credibility of what they say about different things? There were five response categories: “I only trust persons with the same background as my own”, “I trust persons with the same background as my own rather more than others”, “I trust persons with the same background as my own a bit more than others”, “I trust persons with the same background as my own equally as much as others” and “I trust persons with the same background as my own less than others”. The first three categories were dichotomized as “dominant bonding trust” and the last two categories as “dominant bridging trust”.

Bonding vs. bridging relationships were assessed by the question “Who do you socialize most with, people of Swedish origin or with people with a foreign background?” with four response categories: “mostly with Thai people”, “mostly with Swedish people”, “mostly with people who have another foreign background” and “about the same with all groups”. The first category was dichotomized into “dominant bonding relationships” and the three others into “dominant bridging relationships”.

Social participation was measured with the questions “Have you participated in any of the following activities in the last 12 months?” with 13 different social activities and a score ranging from 0 to 13. Based on the median score, respondents who participated in ≤2 (under the median) activities were dichotomized into “low social participation” and those with ≥3 (above the median) activities into “high social participation”.

### Data analysis

The data were analysed with IBM SPSS version 22, with which two different statistical analyses were performed. Bivariate logistic regression was performed to calculate the crude odds ratios (OR), with 95 % confidence intervals, to estimate the extent that various explanatory variables affected the outcomes variables. Multivariate logistic regression with step-wise, two-step models for the potential confounders was used to investigate adjusted associations between the explanatory variables and outcome variables. In the first model for knowledge about sexual and reproductive healthcare services, we included age, marital status and education level; the final model (model 2) included the same variables as model 1 plus socio-economic status and social capital. The first model for contraceptive knowledge was the same as model 1 for knowledge about reproductive healthcare services; the final model (model 2) was also the same as the previous model 2, but we added utilization of healthcare services for contraceptive knowledge. The same models were used for utilization of healthcare services, but in model 2 we included knowledge about reproductive healthcare services. The results are presented in tables including frequencies, percentages and also as crude and multivariate odds ratios (OR) with 95 % confidence intervals (CIs) and p-values. A p-value of <0.05 was considered statistically significant for all statistical analyses.

In order to examine the internal validity of the knowledge questions about healthcare services we analysed those who did not knew where to go, whether they utilize the corresponding healthcare services. The following items were included in the analysis: 1) “Do you know where to go if you would like to take a HIV/STI test?” and “Have you ever been in contact with healthcare services in Sweden to get tested for chlamydia/HIV/hepatitis?”, 2) “Do you know where to go if you want advice on contraception?” and “Have you ever been in contact with healthcare services in Sweden to get advice on contraception?”, and 3) “Do you know where to go if you want to get a gynaecological screening?”, and “Have you ever been in contact with healthcare services in Sweden to get a gynaecological screening?”. The analysis showed that very few (4.2 % had received advice on contraception and 5.3 % had been tested for chlamydia/HIV/hepatitis) utilized the corresponding healthcare, with one exception where 33 % had actually done a gynaecological despite lack of knowledge about where to go if they need such healthcare. In Sweden, all women aged 23–60 are regularly called on to undergo a cervical cancer screening.

## Results

### Socio-demographic characteristics and social capital

The socio-demographic characteristics and social capital of 804 Thai women are summarised in Table 1. The average age was 37 years, where most of the women were in the age group 31–45 years (60.4 %). About half of the women (45.5 %) received their Swedish residence permits in 2006–2007. The majority (85.4 %) of the women were married or cohabiting. Of these, more than two-thirds (72 %) had a Swedish partner (data not shown). Slightly more than one-third (36.0 %) had a low level of education, from 0–6 years. The majority (86.2 %) had attended a Swedish language school. Three out of four (74.5 %) reported lacking cash reserves, meaning that they would not have been able to get hold of 15,000 SEK within a week if needed. Nearly half of the women (45.9 %) reported low trust in others. Approximately 64 % reported dominant bridging trust (as opposed to bonding) and about as many reported dominant bridging relationships (as opposed to bonding). Almost half of the women (48.4 %) had low social participation.

**Table 1:** Respondents’ socio-demographic characteristics and social capital (N = 804)

Variables	*N* (%)
Age	
18–30	177 (22.0)
31–45	486 (60.4)
46 +	141 (17.5)
Total	804
Married/cohabiting	
Yes	618 (85.4)
No	106 (14.6)
Total	724
Education level	
0–6 years	271 (36.0)
7–9 years	121 (16.0)
10–12 years	146 (19.4)
More than 12 years	215 (28.6)
Total	753
Immigration year	
2010–2011	120 (16.0)
2008–2009	290 (38.5)
2006–2007	342 (45.5)
Total	752
Participation in Swedish language School	
Yes	667 (80.0)
No	103 (12.8)
Total	770
Lacking cash reserves^a^	
Yes	178 (22.1)
No	599 (74.5)
Total	777
Trust in others	
Low	320 (45.9)
High	377 (54.1)
Total	697
Bonding vs. bridging trust	
Dominant bonding trust	240 (36.2)
Dominant bridging trust	423 (63.8)
Total	663
Bonding vs. bridging relationships	
Dominant bonding relationships	270 (35.5)
Dominant bridging relationships	490 (64.5)
Total	760
Social participation^b^	
Low	389 (48.4)
High	415 (51.6)
Total	804

^a^not being able to get hold of 15,000 SEK within a week if needed

^b^Low social participation: ≤ 2 activities of 13

### Factors associated with low utilization of healthcare services

Table 2 shows the three different outcomes variables of the study, namely contraceptive knowledge, knowledge about and utilization of sexual and reproductive healthcare services. Utilization of sexual and reproductive healthcare services is based on three questions that are presented in the table. The results show that only one-fourth (25.1 %) of the women had been in contact with healthcare in Sweden to test for HIV/STI. Nearly half (43.3 %) had used healthcare services to get advice on contraception, and about two-thirds (68.7 %) had received a gynaecological screening. When these three questions were recoded into an index score, almost three out of five (58.3 %) of the women were considered to have a low utilization of sexual and reproductive healthcare services (data not shown). The bivariate analysis presented in Table 3, showed that low utilization of healthcare services was significantly associated with the oldest age group (OR = 2.45, CI: 1.42–4.23), living without a partner (OR = 2.26, CI: 1.39–3.67), lacking cash reserves (OR = 1.55, CI: 1.09–2.21), immigration year (OR = 1.68, CI: 1.19–2.36, OR = 2.23, CI: 1.39–3.59), low trust in others (OR = 1.51, CI: 1.09–2.08), having bonding relationships (OR = 1.79, CI: 1.29–2.49) and poor knowledge about reproductive services (OR = 8.14, CI: 5.72–11.57). Results of the multivariate analysis, when adjusted for age, marital status and educational level (model 1), the oldest age group (OR = 2.76, CI: 1.47–5.21) and living without a partner (OR = 2.05, CI: 1.23–3.42) remained associated with low utilization. In the final model (model 2) when adjusted for age, marital status, educational level, socioeconomic status, social capital factors and knowledge of healthcare, living without a partner (OR = 2.53, CI: 1.30–4.90) and poor knowledge of sexual and reproductive healthcare services (OR = 6.07, CI: 3.94–9.34) were significantly related to low utilization. No association was found between education and utilization of healthcare.

**Table 2:** Contraceptive knowledge, knowledge and utilization of sexual and reproductive healthcare services

	Yes		No		Total
	*N*	(%)	*N*	(%)	*N*
Contraceptive knowledge					
Do you know how to protect yourself from an unwanted pregnancy?	696	(91.6)	64	(8.4)	760
Do you know how to protect yourself from HIV/STI?	676	(91.1)	66	(8.9)	742
Knowledge about sexual and reproductive healthcare services					
Do you know where to go if you get pregnant and are considering an abortion?	302	(40.8)	438	(59.2)	740
Do you know where to go if you want to take a HIV/STI test?	293	(41.0)	421	(59.0)	714
Do you know where to go if you want advice on contraception?	458	(62.5)	275	(37.5)	733
Do you know where to go if you want to get a gynecological screening?	653	(86.4)	103	(13.6)	756
Utilization of sexual and reproductive healthcare services					
Have you ever been in contact with healthcare services in Sweden to get a gynecological screening?	524	(68.7)	239	(31.3)	763
Have you ever been in contact with healthcare services in Sweden to get advice on contraception?	315	(43.3)	413	(56.7)	723
Have you ever been in contact with healthcare services in Sweden to get tested for chlamydia/HIV/hepatitis?	178	(25.1)	532	(74.9)	701

**Table 3:** Odds ratios (ORs), factors associated with low utilization of healthcare

Variables		Crude OR (95 % CI)		Model 1 OR (95 % CI)		Model 2 OR (95 % CI)	
	Number of respondents/analysis			Number of respondents/analysis: 605		Number of respondents/analysis: 472	
Age	696						
18–30		1		1		1	
31–45		0.81	(0.56–1.17)	0.91	(0.60–1.38)	1	(0.58–1.70)
46 +		2.45	(1.42–4.23)**	2.76	(1.47–5.21)**	2.11	(0.95–4.70)
Married/cohabating	635						
Yes		1		1		1	
No		2.26	(1.39–3.67)**	2.05	(1.23–3.42)**	2.53	(1.30–4.90)**
Education level	658						
0–6 years		0.82	(0.55–1.21)	0.74	(0.49–1.13)	0.68	(0.39–1.18)
7–9 years		0.78	(0.48–1.26)	0.82	(0.49–1.36)	0.94	(0.50–1.75)
10–12 years		0.62	(0.40–0.96)	0.64	(0.40–1.02)	0.66	(0.37–1.17)
More than 12 years		1		1		1	
Immigration year	654						
2010–2011		2.23	(1.39–3.59)**				
2008–2009		1.68	(1.19–2.36)**				
2006–2007		1				1	
Lacking cash reserves	677						
No		1				1	
Yes		1.55	(1.09–2.21)*	1		1.27	(0.79–2.05)
Trust in others	629						
Low		1.51	(1.09–2.08)*			1.22	(0.79–1.87)
High		1		1		1	
Bonding vs. bridging trust	601						
Dominant bonding trust		1.01	(0.72–1.42)			0.68	(0.43–1.07)
Dominant bridging trust		1		1		1	
Bonding vs. bridging relationships	598						
Dominant bonding relationships		1.79	(1.29–2.49)**			1.56	(1–2.43)
Dominant bridging relationships		1		1		1	
Social participation	696						
Low		1.20	(0.88–1.62)			1.16	(0.75–1.80)
High		1		1		1	
Knowledge about sexual and reproductive healthcare services	665						
Poor		8.14	(5.72–11.57)***			6.07	(3.94–9.34)***
Good		1				1	

Crude OR for considered explanatory factors. Model 1 Adj. OR for included explanatory factors: age, marital status, education. Model 2 Adj. OR for included explanatory factors in model 1 + economic status, social capital, knowledge about sexual and reproductive healthcare services

*P < 0.05 **P < 0.01 ***P < 0.001

### Risk factors for poor knowledge about sexual and reproductive healthcare services

More than half of the women (52.1 %) were classified as having poor knowledge of where to go if they needed to use any of the sexual and reproductive healthcare services in Sweden, and the remaining women (47.9 %) were classified as having good knowledge (data not shown). Four questions were related to this variable of poor/good knowledge and are presented in Table 2. The bivariate analysis identified nine of the ten variables as risk factors for having poor knowledge (Table 4). The oldest age group (OR = 2.77, CI: 1.64–4.66), women with no partner (OR = 1.61, CI: 1.03–2.52), immigration year 2008–2009 (OR = 2.03, CI: 1.45–2.86), 2010–2011 (OR = 2.41, CI: 1.53–3.81) and those who reported lacking cash reserves (OR = 1.78, CI: 1.25–2.54) were more likely to have poor knowledge of sexual and reproductive healthcare services. Social capital factors were other significant predictors of poor knowledge. Women with low trust in others (OR = 1.50, CI: 1.10–2.06) and low social participation (OR = 1.54, CI: 1.14–2.08), and those who reported dominant bonding trust in others (OR = 1.50, CI: 1.10–2.17) and dominant bonding relationships (OR = 1.80, CI: 1.31–2.48), were also more likely to have poor knowledge. The level of education did not influence women’s knowledge. However, results of the multivariate analysis in the fully adjusted model (model 2) showed that the only risk factors for poor knowledge are oldest age group (OR = 2.65, CI: 1.32–5.29), women without a partner (OR = 2.02, CI: 1.16–3.54), women who reported low trust in others (OR = 1.61, CI: 1.10–2.35) and those who reported dominant bonding relationships (OR = 1.50, CI: 1.02–2.23).

**Table 4:** Odds ratios (ORs), factors associated with poor knowledge about sexual and reproductive healthcare services

Variables		Crude OR (95 % CI)		Model 1 OR (95 % CI)		Model 2 OR (95 % CI)	
	Number of respodents/analysis			Number of respodents/analysis:607		Number of respodents/analysis:485	
Age	700						
18-–30		1		1		1	
31–45		0.96	(0.67–1.38)	1.04	(0.69–1.57)	1.10	(0.69–1.78)
46 +		2.77	(1.64–4.66)***	3.1	(1.69–5.66)***	2.65	(1.32–5.29)**
Married/cohabating	640						
Yes		1		1		1	
No		1.61	(1.03–2.52)*	1.66	(1.03–2.69)*	2.02	(1.16–3.54)*
Education level	658						
0–6 years		1.01	(0.69–1.48)	0.87	(0.57–1.31)	0.70	(0.43–1.15)
7–9 years		0.9	(0.55–1.41)	0.99	(0.60–1.64)	0.89	(0.51–1.57)
10–12 years		1.13	(0.73–1.74)	1.18	(0.74–1.86)	1.26	(0.75–2.12)
More than 12 years		1		1		1	
Immigration year	658						
2010–2011		2.41	(1.53–3.81)***				
2008–2009		2.03	(1.45–2.86)***				
2006–2007		1					
Lacking cash reserves	681						
No		1				1	
Yes		1.78	(1.25-–2.54)**			1.50	(0.97–2.32)
Trust in others	632						
Low		1.50	(1.10–2.06)*			1.61	(1.10–2.35)*
High		1				1	
Bonding vs. bridging trust	600						
Dominant bonding trust		1.50	(1.10–2.17)*			1.37	(0.92–2.03)
Dominant bridging trust		1				1	
Bonding vs. bridging relationships	598						
Dominant bonding relationships		1.80	(1.31–2.48)***			1.50	(1.02–2.23)*
Dominant bridging relationships		1				1	
Social participation	700						
Low		1.54	(1.14–2.08)*			1.41	(0.96–2.08)
High		1				1	

Crude OR for considered explanatory factors

Model 1 Adj. OR for included explanatory factors: age, marital status, education

Model 2 Adj. OR for included explanatory factors in model 1 + economic status, social capital

*P < 0.05 **P < 0.01 ***P < 0.001

### Contraceptive knowledge: preventing unwanted pregnancy and STI

Two questions were related to contraceptive knowledge: the women were asked if they knew how to protect themselves from unwanted pregnancy and sexually transmitted infections (STI). Contraceptive knowledge was generally high among the women (Table 2). A large majority reported that they had knowledge of how to protect themselves from an unwanted pregnancy (91.6 %) and of protection against HIV/STI (91.1 %). Results of the bivariate analyses in Table 5 showed that contraceptive knowledge in terms of how to protect from an unwanted pregnancy was significantly associated with education level, age and utilization of sexual and reproductive healthcare services. Women with the lowest education level (OR = 3.78, CI: 1.77–8.06) were more likely to lack knowledge compared to those with more than 12 years of education. The oldest age group (OR = 4.79, CI: 1.97–11.68) knew significantly less than the youngest group. Women with low utilization of healthcare services (OR = 2.72, CI: 1.44–5.14) knew significantly less than those who were considered to have an average utilization. At the multivariate level in the fully adjusted model (model 2), oldest age group (OR = 4.36, CI: 1.03–18.36), those with the lowest education level (OR = 3.73, CI: 1.43–9.73) and low utilization of healthcare services (OR = 2.63, CI: 1.05–6.56) remained associated with a lack of knowledge (Table 5). Regarding a lack of knowledge as to protecting oneself from HIV/STI (Table 6), this outcome was also significantly associated at the bivariate level with low education level (OR = 5.62, CI: 2.45–12.9), oldest age group (OR = 3.30, CI: 1.44–7.57) and low utilization of healthcare services (OR = 2.04, CI: 1.13–3.71). In the final model (model 2), when adjusted for economic status, social capital and utilization of healthcare services, only women with the lowest level of education (OR = 5.29, CI: 1.82–15.34) were more likely to have a lack of knowledge. Marital status, immigration year, economic status and social capital factors did not influence Thai women’s knowledge of preventing an unwanted pregnancy and HIV/STI.

**Table 5:** Odds ratios (ORs), factors associated with lack of knowledge about preventing unwanted pregnancy

Variables		Crude OR (95 % CI)		Model 1 OR (95 % CI)		Model 2 OR (95 % CI)	
	Number of respondents/analysis			Number of respondents/analysis:655		Number of respondents/analysys:480	
Age	760						
18–30		1		1		1	
31–45		1.95	(0.85–4.46)	1.42	(0.56–3.63)	1.76	(0.47–6.67)
46 +		4.79	(1.97–11.68)**	3.3	(1.17–9.32)*	4.36	(1.03–18.36)*
Married/cohabiting	690						
Yes		1		1		1	
No		0.75	(0.31–1.80)	0.82	(0.33–2.04)	0.78	(0.25–2.48)
Education level	715						
0–6 years		3.78	(1.77–8.06)**	3.39	(1.55–7.43)**	3.73	(1.43–9.73)**
7–9 years		1.44	(0.52–3.96)	1.56	(0.53–4.58)	1.89	(0.51–7.04)
10–12 years		1.17	(0.43–3.22)	1.13	(0.39–3.26)	0.48	(0.21–3.39)
More than 12 years		1		1		1	
Immigration year	716						
2010–2011		0.92	(0.44–1.95)				
2008–2009		0.82	(0.46–1.47)				
2006–2007		1					
Lacking cash reserves	740						
No		1				1	
Yes		1.67	(0.83–3.37)			1.33	(0.47–3.75)
Trust in others	673						
Low		0.98	(0.55–1.77)			0.95	(0.43–2.13)
High		1				1	
Bonding vs. bridging trust	642						
Dominant bonding trust		1.01	(0.54–1.87)			1.08	(0.48-–2.45)
Dominant bridging trust		1				1	
Bonding vs. bridging relationships	723						
Dominant bonding relationships		1.17	(0.67–2.04)			0.70	(0.30–1.63)
Dominant bridging relationships		1				1	
Social participation	760						
Low		1.87	(1.11–3.15)*			1.24	(0.56–2.76)
High		1				1	
Use of sexual and reproductive healthcare services	689						
Low utilization		2.72	(1.44–5.14)**			2.63	(1.05–6.56)*
High/average utilization		1				1	

Crude OR for considered explanatory factors. Model 1: Adj. OR for included explanatory factors: age, marital status, education. Model 2: Adj. OR for included explanatory factors in model 1 + economic status, social capital, use of healthcare

*P < 0.05 **P < 0.01 ***P < 0.001

**Table 6:** Odds ratios (ORs), factors associated with lack of knowledge about preventing HIV/STI

Variables		Crude OR (95 % CI)		Model 1 OR (95 % CI)		Model 2 OR (95 % CI)	
	Number of respondents/analysis			Number of respondents/analysis:641		Number of respondents/analysis:477	
Age	742						
18–30		1		1		1	
31–45		1.65	(0.78–3.49)	1.14	(0.49–2.64)	1.94	(0.53–7.07)
46 +		3.30	(1.44–7.57)**	2.18	(0.82–5.76)	3.20	(0.74–13.88)
Married/cohabiting	675						
Yes		1		1		1	
No		0.57	(0.22–1.46)	0.54	(0.21–1.44)	0.59	(0.17–2.11)
Education level	697						
0–6 years		5.62	(2.45–12.9)***	5.21	(2.22–12.21)***	5.29	(1.82–15.34)**
7–9 years		2.45	(0.89–6.76)	2.69	(0.93–7.73)	2.46	(0.62–9.73)
10–12 years		2.23	(0.83–6.0)	1.68	(0.57–4.94)	1.95	(0.54–6.99)
More than 12 years		1		1		1	
Immigration year	702						
2010–2011		0.85	(0.4–1.8)				
2008–2009		0.74	(0.41–1.33)				
2006–2007		1					
Lacking cash reserves	721						
No		1				1	
Yes		1.35	(0.70–2.60)			0.89	(0.35–2.26)
Trust in others	663						
Low		0.84	(0.45–1.55)			0.94	(0.42–2.09)
High		1				1	
Bonding vs. bridging trust	631						
Dominant bonding trust		0.66	(0.40–1.27)			0.72	(0.30–1.72)
Dominant bridging trust		1				1	
Bonding vs. bridging relationships	708						
Dominant bonding social relationships		1.03	(0.59–1.80)			0.77	(0.33–1.82)
Dominant bridging social relationships		1				1	
Social participation							
Low		1.53	(0.92–2.55)			0.75	(0.33–1.69)
High		1				1	
Use of sexual and reproductive healthcare services	684						
Low utilization		2.04	(1.13–3.71)*			1.93	(0.83–4.53)
High/average utilization		1				1	

Crude OR for considered explanatory factors. Model 1: Adj. OR for included explanatory factors: age, marital status, education

Model 2: Adj. OR for included explanatory factors in model 1 + economic status, social capital, use of healthcare services

*P < 0.05 **P < 0.01 ***P < 0.001

## Discussion

The purpose of this study was to investigate the association between knowledge and utilization of sexual and reproductive healthcare services, contraceptive knowledge and socio-demographic characteristics and social capital among Thai immigrant women in Sweden. The findings of the study indicate that some of the social capital factors promote access to knowledge and utilization of the Swedish healthcare services among Thai women in Sweden. Women who reported low trust in others and dominant bonding relationships had lower utilization of healthcare services than those with high trust and dominant bridging relationships. These findings are similar to those reported in previous research, where people with high social capital have better access to healthcare services [29, 50]. Socio-demographic factors also influenced Thai women’s utilization of healthcare services. Low utilization of healthcare was significantly associated with the oldest age group, women with no partner and those who reported lacking cash reserves. Similar results have been found in previous research, where persons in low income groups, persons living without a partner and women of non-Swedish origin were more likely to refrain from medical care [5, 51]. The fact that older women in our study used sexual and reproductive healthcare services less than the younger age groups seems reasonable, since they probably did not have the same need of such healthcare services.

Furthermore, as shown in previous research [46], those with poor knowledge about sexual and reproductive healthcare services were more likely to have a low utilization than those with good knowledge. In our study, women with a lack of knowledge about preventing an unwanted pregnancy reported low utilization of healthcare services more often than those with knowledge. A study done in Sweden found that young immigrant women had less experience of use of contraceptives and contraceptive counselling than young ethnic Swedish women [52]. Immigration year was another important factor for knowledge about and utilization of healthcare services. Earlier research has shown that a longer length of residence increases immigrants’ participation in cervical cancer screening [53] and level of knowledge about HIV/AIDS [54]. In our study, women with a longer length of residence in Sweden reported better knowledge of sexual and reproductive healthcare services and a higher utilization of healthcare services. In the final adjusted model, living without a partner and poor knowledge remained statistically significantly associated with low utilization. Nevertheless, low utilization of healthcare services does not necessarily mean that the women did not have access to HIV preventive measures since condoms are available at most public places in Sweden. However, to what extent condoms were used by the participants was not in the scope of the study.

Social capital factors were also significantly associated with knowledge about Swedish sexual and reproductive healthcare services. Low trust in others and low social participation, dominant bonding trust and bonding social relationships were significantly associated with poor knowledge about the Swedish healthcare services. However, in the final model after controlling for potential confounders, only low trust in others and dominant bonding relationships remained significantly associated with poor knowledge. This result indicates that social relationships with heterogeneously ethnic people is of importance for access to such knowledge about the Swedish healthcare services. No comparable studies have been found. However, our findings support some of the mechanisms that explain the association between social capital and health outcomes, in that social relationships develop channels for the distribution of knowledge and information [28] and thus heterogeneous relationships enhance access to external resources [55]. In the final adjusted model, we also found that women in the oldest age group and women living without a partner were significantly associated with poor knowledge. The fact that women with a partner had better knowledge than women without a partner can possibly be explained by the partner being the primary source of information concerning the healthcare system. As more than half of the women (52.1 %) reported having poor knowledge of where to turn when in need of sexual and reproductive healthcare services, it is recommended that Thai women should receive more information about the Swedish sexual and reproductive healthcare services. They should also be offered society orientation comparable to what immigrants are currently offered when they study at the Swedish language school. According to Swedish law (2013:156) [56], every municipality is obliged to offer society orientation to certain newly arrived immigrants (such as refugees and asylum-seekers) with a residence registration in the municipality.

The descriptive results are also important findings, highlighting that only 25 % of the Thai women reported that they had been HIV/STI tested in Sweden. Research on Thai women’s attitudes towards HIV and HIV testing is needed in order to fully understand this result. It should be noted that a large majority of the women had knowledge of how to protect themselves from HIV/STI. However, knowledge does not necessarily lead to reduction in high-risk sexual behaviour [57]. Furthermore, the increasing number of Thai women immigrating to Sweden and the HIV prevalence among immigrants from Thailand makes it important to study Thai women in order to understand how the Swedish healthcare services can be made more available to them. There is currently no HIV prevention that addresses the Thai group [58]. As Thai women are not included in health examinations, there is also a risk for hidden cases of HIV among Thai immigrants in Sweden and therefore a risk that they are unable to access treatment at an early stage. To improve the availability of HIV testing among these women, they should also be offered a free health examination, in the same way that refugees and asylum-seekers are. Access to free health examinations could also improve Thai women’s knowledge about the Swedish healthcare services, since one of the intentions of the health examination is to introduce migrants to the Swedish healthcare system.

Our findings in this study highlight the need to expand research and interventions among Thai women. Policy makers and healthcare professionals should be aware of Thai women’s marginalized situation in Sweden. Last but not least, future research should include healthcare professionals and staff from other government bodies who meet Thai women in order to obtain better knowledge for possible interventions directed to this group.

## Strengths and limitations

There are several limitations in this study that need to be mentioned. First, this is a cross-sectional study, thus limiting the possibilities of drawing conclusions on causal relationships. Thus, the models used cannot predict the respondents’ knowledge and utilization of healthcare. The models used in this study are based on our assumption that having knowledge about healthcare services provides access to the utilization of healthcare, and utilization of healthcare services may enhance contraceptive knowledge. Second, the outcome measures are based on self-reported knowledge, and the data reported is therefore of a subjective nature. For example, some respondents might think that they have knowledge, and therefore there is a risk of an overestimation of knowledge or overconfidence about incorrect knowledge. It has been shown that individuals with reading problems are often ashamed of this and hide their inability to read [59]. In this study where 36 % of the respondents had 0–6 years of education, this might have led to an overestimation of knowledge. On the other hand, several studies confirm that self-reported sexual behavior and HIV/STI testing have been underreported in relation to actual HIV/STI testing [60, 61], limited by a social desirability bias [62]. However, our analysis showed that few of those who were considered to have poor knowledge of healthcare services and contraceptive knowledge had actually not utilized such services. Also, the outcome measure “utilization of sexual and reproductive healthcare services” was based on self-reported use of healthcare services. Thus, it does not measure respondents’ actual perceived need of sexual and reproductive healthcare, and it may therefore be difficult to interpret the results of this measurement. However, similar measurement instruments on utilization of healthcare have been used in various health surveys, and previous research has shown that self-reporting offers a reasonably valid estimate of differences in the use of healthcare between socioeconomic groups [63]. Third, even though the questionnaire was pre-tested in focus groups and had gone through an extensive translation process, some of the questionnaires were primary developed for the purpose of this specific study and might need further adjustment for use among other ethnic groups. Fourth, a potential limitation of the study was the target population, which consisted of Thai women in the southern part of Sweden only. This group may not have the same experience of healthcare use as Thai women living in more rural areas (more rural areas than in the population selected here), such as northern Sweden, because of the longer distance in the north to healthcare services. It is also possible that Thai women in more rural areas would have more limited social networks and lower social participation. Furthermore, Thai women who resided in Sweden before 2006 would probably have better social networks and higher social participation, and also a higher use of healthcare services, than the women in our study.

One of the strengths of this study is that the response rate was 62.3 %, which is higher than similar investigations such as the Swedish Regional or National Survey of Public Health [64]. This relatively high response rate can probably be explained by the questionnaire being in the Thai language, which made it possible for all Thai women who are able to read and write to answer it, regardless of their Swedish language skills. However, even though the questionnaire was in Thai, there might be some Thais who are illiterate and therefore not able to answer the questionnaire. This could possibly explain the 37.7 % non-response rate.

Despite the relatively high response rate, there were internal missing values for several questions. Hence, this might be mentioned as a limitation for the multivariate analysis because of the reduction of the sample size in the models. However, this does not seem to affect the precision of the estimates since the confidence intervals remained relatively constant in both bivariate and multivariate analysis where associations were found. Another strength of the study is that it was possible to control for important potentially confounding factors, e.g. age, marital status and level of education.

## Conclusion

This study shows that the majority of Thai women had poor knowledge of where to turn when they needed sexual and reproductive healthcare services. Social capital, measured in terms of trust in others and bonding vs. bridging relationships, was of importance for such knowledge. The majority also had a low utilization of sexual and reproductive healthcare services. Only one-fourth reported that they had been HIV/STI tested. Due to HIV prevalence among Thai immigrants in Sweden, Thai immigrants should be entitled to the same type of statutory health examination as refugees and asylum-seekers are. To achieve the goal of “healthcare on equal terms”, policy makers and health professionals must include Thai immigrants when they plan health promotion efforts and healthcare interventions.

## Abbreviations

RTB: Total population register
STI: Sexually transmitted infections
